# Association of ABO and Rh Blood Group Phenotypes with Type 2 Diabetes Mellitus at Felege Hiwot Comprehensive Referral Hospital Bahir Dar, Northwest Ethiopia

**DOI:** 10.1155/2020/2535843

**Published:** 2020-11-05

**Authors:** Biruk Legese, Molla Abebe, Alebachew Fasil

**Affiliations:** ^1^Infectious Disease Screening Division, Amhara National Regional State Health Bureau, Bahir Dar Blood Bank Laboratory, Bahir Dar, Ethiopia; ^2^Department of Clinical Chemistry, School of Biomedical and Laboratory Sciences, College of Medicine and Health Sciences, University of Gondar, Gondar, Ethiopia

## Abstract

**Background:**

ABO and Rh blood group antigens are thought to be among genetic determinants of type 2 diabetes mellitus. Identification of blood group phenotypes are more associated with type 2 diabetes mellitus. It will be helpful for individuals who are susceptible blood groups to take care of themselves by avoiding other predisposing factors and taking preventive measures.

**Methods:**

Hospital-based comparative cross-sectional study was carried out from February to April 2019 at Felege Hiwot Comprehensive Referral Hospital. Sociodemographic and clinical data were collected with a semistructured pretested questionnaire. ABO and Rh Blood group were determined by slide and test tube methods. Biochemical parameters were determined with Mindray BS-200E fully automated clinical chemistry analyzer. Data were analyzed by IBM SPSS version 20 statistical software. Chi-square test and logistic regression analysis were employed for data analysis. A *P* value of < 0.05 was considered statistically significant.

**Results:**

From a total of 424 participants included for this study, blood group O was found higher in frequency with 74 (34.9%) and 97 (45.75%) for cases and healthy controls, respectively. ABO blood groups showed significant association with T2DM, a chi-square value of 12.163 and *P* value of 0.007. However, the Rh blood group was not associated with T2DM. Binary logistic regression analysis revealed that blood group B had a higher risk (OR: 2.12, 95% CI: 1.33-3.32) and blood group O had decreased risk (OR: 0.636, 95% CI: 0.43-0.94) of T2DM as compared to other blood groups.

**Conclusion:**

ABO blood group antigens showed significant association with type 2 diabetes mellitus. Blood group B was associated with an increased risk and O blood group with decreased risk of type 2 diabetes mellitus.

## 1. Introduction

Diabetes mellitus (DM) is a metabolic disorder of multiple etiologies characterized by chronic hyperglycemia with disturbances of carbohydrate, fat, and protein metabolisms resulting from defects in insulin secretion, insulin action, or both [1, 2]. Several pathological processes are involved in the development of DM that range from autoimmune destruction of the *β* cells of the pancreas with consequent insulin deficiency to abnormalities that result in resistance to insulin action [3].

Blood group antigens are thought to be among hereditary determinants and play a vital role to understand genetics and disease susceptibility [4]. Since the discovery of blood groups in 1900, there have been interests to discover a possible association between ABO and Rh blood groups and different diseases [5]. Along with their expression on red blood cells, ABO antigens are also expressed on the surface of many human cells and tissues, including the epithelium, sensory neurons, platelets, and the vascular endothelium [6]. Thus, the clinical significance of the ABO and Rh blood group system extends beyond transfusion medicine, and several studies have suggested an important involvement of the ABO and Rh blood group antigens in the development of different diseases [7, 8]. The data obtained from different studies showed that the ABO and Rh blood group antigens are associated with gastric cancer, salivary gland tumors, duodenal ulcer, colorectal cancer, thyroid disorders, ovarian tumors, coronary heart disease, and DM especially T2DM [9–13].

The pathophysiologic mechanisms for the association between ABO blood group phenotypes with T2DM and associated factors are not well understood. However, there are some possible assumptions: the first is that the ABO blood group is linked to specific molecules related to T2DM. Genome-wide association studies documenting that variants at ABO gene loci, especially A and B antigens, are associated with increased levels of plasma lipid and inflammatory markers such as soluble intercellular adhesion molecule 1 (ICAM-1), E-selectin, P-selectin, and tumor necrosis factor-2 (TNF-2), are well known risk factors of DM. These molecules are well-known mediators of inflammation that affects insulin and its receptors and contributed to the development of DM [14, 15].

The ABO gene on chromosome 9q34 encodes glycosyl transferases that catalyze the transfer of nucleotide donor sugars to the H antigen to form the A and B antigens. The transferase enzymes and nucleotide donor sugars also induce the production of inflammatory mediators like interleukin 6 and TNF-*α* in the endothelium [16].

Inflammatory cytokines secreted by endothelium exert an endocrine effect conferring insulin resistance in the liver, skeletal muscle, and vascular endothelial tissue, ultimately leading to the clinical expression of T2DM. These inflammatory markers also lead to an acute phase response with increased hepatic production of C-reactive protein (CRP), a sensitive marker of low-grade systemic inflammation which directly promotes insulin resistance [17].

ABO blood types may also be associated with gut bacteria composition, which may be linked to T2DM. In T2DM, gut dysbiosis contributes to the onset and maintenance of insulin resistance. Different strategies that reduce dysbiosis can improve glycemic control. Evidence in animals and humans reveals the differences between the gut microbial composition in healthy individuals and those with T2DM. Changes in the intestinal ecosystem could cause inflammation, alter intestinal permeability, and modulate metabolism of bile acids, short-chain fatty acids, and metabolites that act synergistically on metabolic regulation systems contributing to insulin resistance [18].

Diabetes mellitus is the most common metabolic disorder affecting people worldwide both in developing and developed countries. People living with DM were estimated to be 451 million in the world by 2017. These figures were expected to increase to 693 million in 2045 [19]. Asia is a major area of the rapidly emerging T2DM global epidemic, with China and India being the top two epicenters [20]. In the African region, the average prevalence was 4.9% in 2013, having Reunion (15.4%), Seychelles (12.1%), and Gabon (10.7%) as the top three countries with higher prevalence [21]. In Ethiopia in 2016, the prevalence of DM was found to be 6.5% [22]. ABO and Rh blood groups are among the genetic factors that contribute to the occurrence of T2DM [23]. The major human blood group systems are ABO and Rh. The frequency distribution of these blood groups varies markedly in different races and ethnic and socioeconomic groups.

Several researches have been conducted to show the association between the ABO and Rh blood groups with T2DM but the results were not consistent and such a study is yet to be conducted in Ethiopia. Therefore, this study is aimed at ascertaining the association of the ABO and Rh blood groups with T2DM among adults with T2DM at Felege Hiwot Comprehensive Referral Hospital, Bahir Dar, Northwest Ethiopia,

## 2. Methods and Materials

### 2.1. Study Area

The study was conducted at FHCRH Diabetic Clinic. The hospital is located in the capital city of Amhara regional state, Bahir Dar, Northwest Ethiopia, about 565 kilometers far away from the capital city of Ethiopia, Addis Ababa. The hospital is one of the biggest hospitals in the Amhara region that provide health services and serves as the referral center for other district hospitals in the region. The hospital is providing services for more than 7 million people.

### 2.2. Study Design and Period

A hospital-based comparative cross-sectional study was conducted from February to April 2019 to assess the association of the ABO and Rh blood groups with DM and associated factors among adults with T2DM attending clinic at FHCRH, Bahir Dar, Ethiopia.

### 2.3. Population

#### 2.3.1. Source Population

All patients served at FHCRH were the source population for cases, and apparently, healthy volunteer blood donors at Bahir Dar Blood bank (BBBS) were the source population for controls.

#### 2.3.2. Study Population

All T2DM patients who had follow-up at FHCRH DM Clinic during the study period that met the eligibility criteria, volunteers, and apparently healthy blood donors who donated blood during the study period at Bahir Dar Blood Bank were included as the study population. During data collection, identification of T2DM from T1DM was done by analyzing the patient's chart.

### 2.4. Sample Size and Sampling Technique

The sample size was determined by using the double population proportion general formula (*N* = 2 × (*p*)(1 − *p*)(*zβ* + (*z*∝)/2)2/(*p*1 − *p*2)2), where *N* was sample size, *p* estimates of the double population proportion, *p*1 proportion ofthe B blood group among healthy controls, *p*2 was the proportion of the B blood group among T2DM patients, *Z*_*β*_ was power, and *Z*_*α*/2_ was the level of significance.

Proportions of the B blood group among DM patients (*p*2 = 33.06%) and apparently healthy controls (*p*1 = 18.62%) from a study done in Malaysia [24], 95% confidence level, 0.05 level of significance, power = 90, and the ratio of controls to cases = 1 were considered and entered into Epi Info version7 software. The total sample size was 424, 212 for each group. Study participants were selected by systematic random sampling technique.

### 2.5. Data Collection Methods

Sociodemographic and clinical data were collected with the semistructured pretested questionnaire by trained nurses. Height and weight of study participants were measured with stadiometer and digital weight scale (Zhongshan Frecom Electronic Company Limited, China), respectively. Blood pressure of the study participants was measured by trained nurses with manual aneroid sphygmomanometer manufactured by Shanghai Caremate Medical Co., Ltd., Shanghai, China.

The ABO blood group of study participants was determined by the slide method using known anti-A and anti-B sera (Spinreact, Spain). The Rh blood group of participants was determined with the slide method and those tested Rh negative were tested again by the test tube method with anti-D and anti-human globulin sera (Spinreact, Spain). Triglyceride (TRG), low-density lipoprotein (LDL), high-density lipoprotein (HDL), total cholesterol (TC), and fasting blood glucose levels of DM patients were determined with Mindray BS-200E (Mindray Medical International Ltd., China). The manufacturer's instruction for each parameter was followed. Glucose, TRG, and TC were determined using glucose oxidase, glycerokinase peroxidase, and cholesterol oxidase peroxidase methods, respectively.

### 2.6. Data Management and Quality Control

The questionnaire was pretested on participants equivalent to 5% of the sample size of the study at the University of Gondar Comprehensive Referral Hospital for its accuracy, consistency, and to estimate the time needed to complete the questionnaire prior to actual data collection. A one-day training was given for data collectors on the objective of the study, consenting, techniques of interview, laboratory test procedures, and their quality control. Data collectors were monitored throughout the whole data collection period. Sociodemographic and clinical data were collected by trained nurses under the supervision of the principal investigator, and the quality of measuring devices was checked daily. In order to assure the quality of the laboratory result, standard operating procedures in preanalytical, analytical, and postanalytical stages were followed. The quality of results was assured by running quality control samples (Humatrol P and Humatrol N) daily. Known A and B cell suspension used to check the quality of anti-A and anti-B sera. The quality control sample results for biochemical profiles were monitored using a Levey-Jennings (LJ) chart.

### 2.7. Data Analysis and Interpretation

The data were checked for completeness, cleaned, arranged, and categorized manually. Then, it was entered and analyzed by SPSS version 20 (IBM, USA). Descriptive statistics were performed for sociodemographic and clinical data, and odds ratio and chi-square values were derived to show the correlation between the ABO blood groups and DM and DM-associated factors. The bivariable logistic and multivariable regression analyses were employed. Variables having a *P* value ≤ 0.2 were incorporated into multivariable logistic regression analysis, and a *P* value < 0.05 was considered statistically significant.

### 2.8. Ethical Considerations

Ethical clearance was obtained from the Research and Ethical Review Committee of the School of Biomedical and Laboratory Sciences, College of Medicine and Health Sciences, University of Gondar. Seal of approval was obtained from FHCRH and BBBS. A full explanation about the purpose of the study was made to the authorized bodies of FHCRH. A permission letter was taken from the medical director of the hospital and head of the DM clinic. To ensure confidentiality of the data, study participants were identified using codes and unauthorized persons had no access to the collected data. Data was collected after full written consent was obtained from each participant. The study was beneficial to study participants as it helped them to know their blood groups and lipid profile level. Abnormal lipid profile results were reported to physicians and nurses at the DM clinic.

## 3. Results

### 3.1. Sociodemographic Characteristics

Males (216) comprised 50.9% of the study participants. The median age of the study participants was 37.4 years (range: 18-89 years). Most of T2DM study participants were married157 (74.1%), self-employed 56 (26.4%), and unable to read and write 100 (47.2%).

### 3.2. Clinical Data of Study Participants

Sixty-five (30.7%) of T2DM cases had a family history of DM. Out of 212 T2DM cases, 4.2%, 26.9%, 62.7%, 63.7%, and 55.7% were cigarette smokers, alcohol drinkers, eat fruits and vegetables sometimes, and did not perform physical exercise, and have normal BMI, respectively. One hundred and sixty-five (77.8%) of T2DM patients had poor glycemic control. Among T2DM patients 51.4%, 62.7%, 78.3%, and 51.4% showed normal TRG, LDL, HDL, and TC, respectively (Table 1).

**Figure 1:**
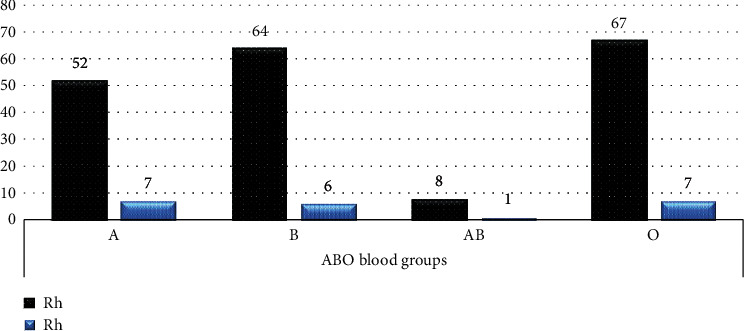
Frequency of the ABO and Rh blood group phenotypes among T2DM patients.

**Figure 2:**
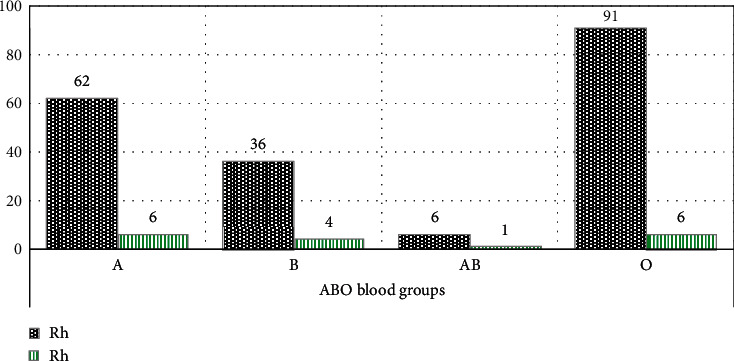
Frequency of the ABO and Rh blood group phenotypes among healthy controls.

Among DM patients, blood group O 74 (34.9%) was the most frequent followed by B 70 (33.0%), A 59 (27.8%), and AB 9 (4.2%), and 191 (90.1%) were Rh ‘D' positive (Figure 1). Among healthy controls blood group O 97 (45.8%) was most frequent followed by A 68 (32.1%), B 40 (18.9%), and AB 7 (3.3%), and 195 (92%) were Rh ‘D' positive (Figure 2).

### 3.3. Association of ABO and Rh Blood Group Phenotypes with T2DM

ABO blood groups were significantly associated with T2DM with a chi-square value of 12.163 and *P* value of 0.007, but Rh blood group was not associated with T2DM (Table 2).

Binary logistic regression analysis indicated that blood group B individuals were 2.12 times more risk to develop T2DM as compared to other ABO blood groups (OR: 2.12; 95% CI: 1.33-3.32). On the other hand, blood group O was protective against T2DM as compared to other blood groups (OR: 0.636; 95% CI: 0.43-0.94). The Rh blood group was not significantly different between the two groups (Table 3).

### 3.4. Factors Associated with ABO and Rh Blood Group Phenotypes

Family history of DM, physical exercise, DBP, BMI, TRG, and HDL cholesterol were not statistically associated with ABO blood group phenotypes. However, SBP (AOR = 2.353, *P* = 0.019) was associated with blood group A; individuals with blood group A were 2.353 times more at risk to be hypertensive as compared to other blood groups. Increased alcohol drinking habit (AOR = 3.362, *P* < 0.0001) and decreased total cholesterol (AOR = 0.496, *P* = 0.029) were associated with blood group B compared with the other blood groups (Table 4).

## 4. Discussion

Many studies have been conducted in order to investigate the possible relationship between the ABO and Rh blood group phenotypes with T2DM and its associated factors. The results have been proved to be inconsistent and differed from one study to another [24–27]. The results of the present study supported the assumption that ABO blood group phenotypes are associated with the risk of developing T2DM. Our finding was similar with studies done in Saudi Arabia [28] and Malaysia [24, 29]. Contrary to the current findings, studies conducted in India [30], Iran [27], and Algeria [26] reported nonstatistically significant association between DM and any of ABO blood group phenotypes. The possible reason for this contradiction might be sample size, age and gender distribution, and a difference in racial and environmental factors which may affect the distribution of ABO blood group phenotypes and disease occurrence [23].

Findings of the current study revealed that study participants with blood group B were more affected by T2DM as compared with healthy controls. The rationales behind this observed association might be the existence of higher levels of inflammatory mediators like factor VIII-VWF complex, ICAM-1, and TNF-2 in blood group B individuals. It is well stated that systemic inflammation is the main cause of insulin resistance and ultimately plays a role in the development of T2DM [31, 32]. Similar results were reported by studies in Qatar [25], Saudi Arabia [28], India [30, 33, 34], Malaysia [24], and France [23]. However, a study conducted in Pakistan [5] indicated that blood groups B and A were less likely to develop p to T2DM as compared to other blood groups. The possible justifications for the observed difference may be geographical and racial differences which may affect the genetic expression of disease and the frequency of ABO blood group antigens [33].

Blood group ‘O' individuals were less likely to develop T2DM compared to other ABO blood types. In line with this study, studies in Qatar [25], Saudi Arabia [28], Malaysia [29], and India [35] reported a similar result. A study in Iran [36] also showed a decreased risk of O blood group to develop DM, but the association was not statistically significant. The reason for this protective effect of the O blood group might be the low level of inflammatory mediators like factor VIII-VWF complex, intercellular adhesion molecule-1 (ICAM-1), and TNF-2 [31, 32]. Inconsistent to the current result, a study in India [37] reported that blood group O had increased risk of developing DM as compared to other blood groups. The reasons for the observed difference might be the geographical, environmental, and genetic differences in which these studies were conducted.

In the current study, even though the frequency of the A blood group was slightly higher among controls (*n* = 68) than DM patients (*n* = 59), it did not show statistically significant association with T2DM. Concordant results to the current finding were reported in Qatar [25] and Malaysia [38]. However, studies in Pakistan [5], Malaysia [35], and Egypt [39] revealed that blood group A was significantly associated with T2DM.

From this study, blood group AB was not associated with T2DM. However, a study done in Egypt [39] showed that blood group AB was protective against T2DM, and another study in India [37] showed that the AB blood group was higher among T2DM patients as compared to healthy controls. The reasons for this variation might be the geographical, genetic, and environmental difference in the study area.

The result of the present study showed that the Rh factor was not associated with T2DM. Similar results were reported by studies in India [37] and Algeria [26]. However, a study conducted in Pakistan [5] indicated that Rh-negative blood groups and T2DM had a significant association. On the other side, research done in Iran [40] showed that Rh-positive blood groups are positively associated with T2DM.

In our study, O+ and B+ blood groups were significantly associated with the risk of T2DM. In support of the present study, a study in France [23] reported that blood group B+ showed a higher risk for DM; another study from Nigeria [41] conducted on both types of diabetes types reported that O+ blood group was significantly lower in diabetics patients than in the control population.

In the current study, ABO blood groups showed an association with DM-associated factors. Blood group A was significantly and positively associated with hypertension (increased systolic blood pressure) as compared to other blood groups. Studies conducted in Bosnia and Herzegovina [42] and India [43] among African-origin populations also reported similar findings to the current finding. Discordant to our results, a study conducted in Egypt [39] revealed that blood group B had a significantly higher risk for hypertension. Studies conducted in Iran [40] and India [44] also attested different results compared to the current study, in which individuals with blood group B had significantly increased risk of developing hypertension.

The result of our study revealed that blood group B was associated with a decreased level of total cholesterol as compared to other blood groups. Contrary to the current results, a study in Egypt demonstrated that individuals with blood group B showed a significant elevation in total cholesterol and triglyceride levels [39]. Another research in Nigeria also revealed that blood group A individuals had increased levels of LDL cholesterol as compared to other blood groups [45]. The current study also showed an association between alcohol drinking habits with ABO blood groups. In this case, blood group B was associated with alcohol drinking habit in DM patients. In line with our result, a study in Nigeria [46] revealed that alcohol drinking habit was associated with ABO blood groups.

The mechanisms for the observed association between ABO blood group phenotypes with T2DM are still unknown. There are some possible suggested assumptions. The first suggestion was that the human ABO antigen locus might influence inflammatory mediators, such as factor VIII-von Willebrand factor (VWF) complex, which is present in higher levels in non-O individuals [31]. In addition, ABO blood group antigens have been linked with plasma-soluble ICAM-1 and TNF-R levels. These both markers have been associated with an increased type 2 diabetes risk thus providing a potential explanation for the observed relationships [32].

## 5. Limitations of the Study

The limitation of this study was the inability to determine the association of ethnic backgrounds with blood groups for the study participants with T2DM.

## 6. Conclusions

From the findings of this study, ABO blood group phenotypes are significantly associated with T2DM. In this study, B blood group was found to be positively associated with T2DM, while O blood group has negative association with T2DM. However, blood groups A, AB, and Rh were not associated with T2DM. This study also sought to determine the relationship between ABO and Rh blood group phenotypes with DM-associated factors, and it was realized that blood group A is associated with an increased systolic BP, and blood group B is significantly associated with decreased levels of total cholesterol.

## Figures and Tables

**Table 1:** Clinical data of DM patients attending at FHCRH, Bahir Dar, Northwest Ethiopia, 2019 (*n* = 212).

Variables		Frequency	Percentage
Family history of DM	Yes	65	30.7
	No	147	69.3
Cigarette smoking habit	Yes	9	4.2
	No	203	95.8
Alcohol drinking habit	Nondrinker	155	73.1
	Light	15	7.1
	Moderate	31	14.6
	Heavy	11	5.2
Eat fruits and vegetables	Do not eat at all	62	29.2
	Some times	133	62.7
	Every day	17	8.0
Physical exercise	Inactive	135	63.7
	Medium	69	32.5
	Highly active	8	3.8
SBP	≤135	125	59.0
	>135	87	41.0
DBP	≤85	146	68.9
	>85	66	31.1
BMI	Underweight	10	4.7
	Normal weight	118	55.7
	Overweight	74	34.9
	Obese	10	4.7
Glycemic control	Good (FBS ≤ 152)	47	22.2
	Poor (FBS > 152)	165	77.8
TRG	≤150 mg/dL	109	51.4
	>150 mg/dL	103	48.6
LDL	≤100 mg/dL	133	62.7
	>100 mg/dL	79	37.3
HDL	≥40 mg/dL	166	78.3
	<40 mg/dL	46	21.7
TC	≤200 mg/dL	109	51.4
	>200 mg/dL	103	48.6

Note: BMI: body mass index; DM: diabetes mellitus; DBP: diastolic blood pressure; HDL: high-density lipoprotein; LDL: low-density lipoprotein; SBP: systolic blood pressure; TRG: triglycerides.

**Table 2:** The association of ABO blood group phenotypes with T2DM at FHCRH, Bahir Dar, northwest Ethiopia, 2019 (*n* = 424).

Blood groups	T2DM patients	Controls	*X* ^2^	*P* value
A	59 (46.5%)	68 (53.5%)	12.163	0.007
B	70 (63.6%)	40 (36.4%)		
AB	9 (56.2%)	7 (43.8%)		
O	74 (43.3%)	97 (56.7%)		
Rh ‘D'+	191 (49.5%)	195 (50.5%)	0.463	0.496
Rh ‘D'-	21 (55.3%)	17 (44.7%)		

**Table 3:** Association of the ABO and Rh blood group phenotypes with T2DM at FHCRH, Bahir Dar, Northwest Ethiopia, 2019 (*n* = 424).

Study group	Non-A	A	COR (95% CI)	*P* value
Control	144 (67.9%)	68 (32.1%)	1	
DM	153 (72.2%)	59(27.8%	0.817 (0.538-1.24)	0.34
	Non-B	B		
Control	172 (81.1%)	40 (18.9%)	1	
DM	142 (67.0%)	70 (33.0%)	2.12 (1.33-3.32)	0.001
	Non-O	O		
Control	115 (54.2%)	97 (45.8%)	1	
DM	138 (65.1%)	74 (34.9%)	0.636 (0.43-0.94)	0.023
	Non-AB	AB		
Control	205 (96.7%)	7 (3.3%)	1	
DM	203 (95.8%)	9 (4.2%)	1.298 (0.474-3.55)	0.611
	Rh+	Rh-		
Control	195 (50.5%)	17 (44.7%)	1	
DM	191 (49.5%)	21 (55.3%)	1.261 (0.645-2.464)	.497
	Non-B+	B+		
Control	176 (83%)	36 (17%)	1	
DM	149 (70.3%)	63 (29.7%)	2.067 (1.300-3.288)	0.002
	Non-O+	O+		
Control	121 (57.1%)	91 (42.9%)	1	
DM	145 (63.4%)	67 (36.6%)	0.614 (0.413-0.914)	0.016

Note: COR: crude odds ratio; DM: diabetes mellitus.

**Table 4:** Association of ABO blood group phenotypes with biochemical and anthropometric measurements at FHCRH, Bahir Dar, northwest Ethiopia 2019 (n =212).

Variables		Non-A	A	COR (95% CI)	AOR (95% CI)	*P* value
SBP	Normal	99 (79.2%)	26 (20.8%)		1	
	Hypertensive	54 (62.1%)	33 (37.9%)	2.327 (1.26-4.29)	2.353 (1.15-4.815)	0.019^∗^
LDL	Normal	91 (68.4%)	42 (31.6%)		1	
	Abnormal	62 (78.5%)	17 (21.5%)	0.594 (0.31-1.137)	0.493 (0.244-0.995)	0.048^∗^
HLD	Normal	122(73.5%)	44 (26.5%)		1	
	Abnormal	31 (67.4%)	15 (32.6%)	1.32 (0.662-2.72)	1.64 (0.774-3.477)	0.197
TC	Normal	83 (76.1%)	26 (23.9%)		1	
	Abnormal	70 (68.0%)	33 (32.0%)	1.5 (0.822-2.75)	1.885 (0.96-3.69)	0.065
		Non-B	B			
SBP	Normal	80 (64.0%)	45 (36.0%)		1	
	Hypertensive	62 (71.3%)	25 (28.7%)	0.72 (0.397-1.294)	0.704 (0.353-1.406)	0.320
DBP	Normal	94 (64.4%)	52 (35.6%)		1	
	Hypertensive	48 (72.7%)	18 (27.3%)	0.678(0.358-1.284)	0.771 (0.377-1.575)	0.475
TC	Normal	65 (59.6%)	44 (40.4%)		1	
	Abnormal	77 (74.8%)	26 (25.2%)	0.499 0,277-0.897)	0.496 (0.265-0.931)	0.029^∗^
Alcohol	No	115 (74.2%)	40 (25.8%)		1	
	Yes	27 (47.4%)	30 (52.6%)	3.19(1.694-6.012)	3.316 (1.728-6.362)	<0.001^∗^

Note: ^∗^Statistically significant association. Abbreviations: Non-A: blood groups other than A; Non-B: blood groups other than B; SBP: systolic blood pressure; DBP: diastolic blood pressure; LDL: low-density lipoprotein; HDL: high-density lipoprotein; TC: total cholesterol.

## Data Availability

The data used to support the findings of this study are available from the corresponding author upon request.
